# Two Mutations in the Caprine *MTHFR* 3'UTR Regulated by MicroRNAs Are Associated with Milk Production Traits

**DOI:** 10.1371/journal.pone.0133015

**Published:** 2015-07-17

**Authors:** Jinxing Hou, Xiaopeng An, Yuxuan Song, Teyang Gao, Yingnan Lei, Binyun Cao

**Affiliations:** College of Animal Science and Technology, Northwest A&F University, Yangling, Shaanxi, 712100, P.R. China; University of Bologna, ITALY

## Abstract

**Background:**

5,10-Methylenetetrahydrofolate reductase (MTHFR) plays a central role in folate metabolism by irreversibly converting 5,10-methylenetetrahydrofolate to 5-methylenetetrahydrofolate, a predominant circulating form of folate. Folate is reportedly important for milk protein synthesis, and MTHFR may be a key regulatory point of folate metabolism for milk protein synthesis in mammary epithelial cells. Prior to this study, polymorphisms of the *MTHFR* gene were not associated with milk production traits from a breeding perspective. Single nucleotide polymorphisms (SNPs) at microRNA (miRNA) binding sites (miR-SNPs) can affect gene expression. This study aimed to identify the effects of miR-SNPs (g.2244A>G and g.2264A>G) in the caprine *MTHFR* 3' UTR on the milk production traits of dairy goats.

**Results:**

Guanzhong dairy (GD, *n* = 325) goats were used to detect SNPs in the caprine *MTHFR* 3' UTR by DNA sequencing. Two novel SNPs (g.2244A>G and g.2264A>G) were identified in the said region. The homozygous haplotype A-G of the SNPs g.2244A>G and g.2264A>G was significantly associated with milk yield and milk protein levels in GD goats (*P* < 0.05). Functional assays indicated that the *MTHFR* 2244 A → G substitution could increase the binding activity of hsa-miR-1266 with the *MTHFR* 3' UTR. The *MTHFR* 2264 A → G substitution could decrease the binding activity of hsa-miR-616 with the *MTHFR* 3' UTR. In addition, we observed a significant increase in the *MTHFR* mRNA levels of homozygous haplotype A-G carriers relative to those of homozygous haplotype G-A carriers. These results indicated that both SNPs altered the *MTHFR* mRNA levels. These altered levels of *MTHFR* mRNA may account for the association of SNPs with milk production traits.

**Conclusions:**

This study is the first to report that the g.2244A>G and g.2264A>G polymorphisms were associated with milk production traits in GD goats. Further investigations should explore the underlying miRNA-mediated mechanisms that are modified by the g.2244A>G and g.2264A>G SNPs. The current study evaluated these SNPs as potential genetic markers in goats, with potential applications in breeding programs.

## Introduction

5,10-Methylenetetrahydrofolate reductase (MTHFR) is an essential enzyme for homocysteine and folate metabolism; this enzyme is involved in one-carbon metabolism and responsible for the final step in the conversion of dietary forms of folate to 5-methyltetrahydrofolate [1–3]. The caprine *MTHFR* gene is located on chromosome 16 and has 13 exons spanning 14.639 kb [4]. Analysis of the genetic variability of *MTHFR* has revealed its metabolic and functional aspects that are relevant to understanding the biology of folate metabolic pathways. The folate metabolism genes (such as *MTHFR*) play critical roles in regulating milk protein synthesis [5]. To the best of our knowledge, the association of *MTHFR* gene polymorphisms with milk production traits has not been reported from an animal breeding perspective.

MicroRNAs (miRNAs) are small RNAs of approximately 21 nucleotides. MiRNA can bind to the 3'untranslated region (3' UTR) of its target mRNA to post-transcriptionally regulate gene expression. Studies have shown that miRNAs play important roles in several biological processes, including embryonic development, cell proliferation and differentiation, apoptosis, fat metabolism, atherosclerosis and oncogenesis [6]. In previous studies, several miRNAs (e.g., miR-200a and miR-21) were found to be involved in the cellular differentiation of mammary glands [7,8].

SNPs are the most abundant form of DNA variation in the animal genome. Brodersen and Voinnet [9] showed that SNPs in miRNA binding sites can affect miRNA-induced genetic repression. SNPs that affect miRNA binding to target genes are called miR-SNPs [10]. Liu et al. [11] reported that the human SNP rs3735590 C → T influences miR-616 binding to the *PON1* gene, thereby increasing the risk of ischemic stroke and carotid atherosclerosis. Clop et al. [12] demonstrated that the *GDF8* allele of Texel sheep is characterised by a G → A transition in the 3' UTR that creates a target site for miR-1 and miR-206, which are highly expressed in skeletal muscle; consequently, translational inhibition of the myostatin gene occurs, thereby contributing to muscular hypertrophy in Texel sheep. Gao et al. [13] showed that the SNP g.1536 C>T in the *TNP2* 3' UTR alters the binding of *TNP2* with bta-miR-154, and is associated with the semen quality of Chinese Holstein bulls. Given the regulatory role of miRNAs in gene expression, miR-SNPs may function as promising markers for milk production traits.

Based on the aforementioned considerations, we detected polymorphisms in the *MTHFR* gene of Guanzhong dairy (GD) goats by DNA sequencing, and investigated the associations between these genetic markers and milk production traits. After evaluating the associations between the candidate miR-SNPs and phenotypes of interest, we conducted reporter assays to confirm the effects of these miR-SNPs.

## Materials and Methods

### Ethics statement

The Animal Ethical Committee at the Northwest A & F University approved the experimental procedures.The animals were reared in GuanzhongDairy Goat Breeding Base in Zhouzhi county of Shaanxi province (34°14'N, 113 108°37'E and 1000 m altitude).

### Animals and genomic DNA isolation

Blood samples were obtained from 325 GD goats. The goats were reared under the same standard conditions with dry-lot nutrition. All animals included in the study descended from 13 GD goat sires. Data on health, fertility and milk production were recorded by dairymen and veterinarians. The milk production traits were recorded from 2008 to 2013. For milk yield analysis, 325 GD goats were milked twice a day with a milking machine throughout the entire lactation period, and the milk weight was measured with an electronic scale. According to Luo [14], milk yield from the first to third lactation periods were standardised to 300 d in milk. For milk constituent analysis, a total of 325 milk samples were collected from the 325 GD goats once a month throughout the full lactation period. The first sampling occurred at least 20 d after parturition to exclude the risk of contamination with colostrum. The milk components (protein and fat) were determined with an ultrasonic S60SEC milk analyser (Milkotronic Company, Nova Zagora, Bulgaria). The consecutive milk records of the same goat were used in the study. For SNP analysis, 5 mL of blood was aseptically collected from the jugular vein of each individual (325 GD goats) and then individually stored in tubes containing the anticoagulant ACD (citric acid: sodium citrate: dextrose, 10:27:38).All samples were transported to the laboratory on ice. Genomic DNA was extracted from white blood cells by standard phenol‒chloroform extraction. All experiments were performed in accordance with the National Institutes of Health Guide for the Care and Use of Laboratory Animals.

### SNP investigation and genotyping

The caprine *MTHFR* gene (GenBank Accession Nos. NC_022308 and XM_005690674) was used to design one pair of primers for amplifying the 3' UTR of caprine *MTHFR* (326bp). The exact position of amplicon in the 3' UTR of caprine *MTHFR* is in 2185bp-2510bp region (GenBank accession No. XM_005690674). Their optimal annealing temperatures are shown in **Table A in S1 File**. Subsequently, we screened the samples to identify SNPs of *MTHFR* by pooled DNA sequencing [15]. One DNA pool was created by mixing 5 μL of DNA (100 ng/μL) from each of the 325 samples. The pooled DNA as a template was used to amplify the 3' UTR of caprine *MTHFR*. A PCR Master Mix (2×) Kit (Thermo Scientific, NY, USA) was used for PCR amplification. The 25 μL PCR reactions contained 50 ng of pooled genomic DNA, 12.5 μL of 2× reaction mix (including 500 μM of each dNTP; 20 mMTris–HCl, pH 9; 100 mMKCl; 3 mM MgCl_2_), 0.5 μM of each primer and 0.5 U of *Taq* DNA polymerase (Thermo Scientific, NY, USA). The cycling protocol involved initial denaturation at 95°C for 5 min, followed by 35 cycles of denaturation at 94°C for 30 s, annealing at 50°C for 30 s and extension at 72°C for 35 s, with a final extension at 72°C for 10 min. Ten PCR products amplified with pooled DNA as a template were sent to the Beijing Genomics Institute (Beijing, China) for Sangersequencing in both directions.SNP discovery was conducted using the Chromas version 2.31 and DNAstar version 7.0 software programs. SNP nomenclature and numbering followed the recommendations of http://www.hgvs.org/mutnomen/recs-DNA.html#number. If SNPs were found in the 3' UTR of caprine *MTHFR*. The 325 samples were amplified independently, and then the 325 PCR products were sequenced independently to detect the different individual genotypes.

### Statistical analysis

Polymorphism information content (PIC) represents the probability that a given offspring gets a certain markeralle from the same markeralle of its father or mother. PIC used to estimate the polymorphism of marker locus.PIC is calculated as follows:

$$PIC=1-\sum_{i=1}^{n}p_{i}^{2}-\sum_{i=1}^{n-1}\;\sum_{j=i+1}^{n}2p_{i}^{2}p_{j}^{2}$$

where *P*_*i*_ and *P*_*j*_ are the allelic frequencies of the i^th^ and j^th^ alleles, respectively, and n is the number of alleles [16].The allele frequencies, heterozygosity (He) and PIC were calculated using POPGENE (version 1.31; http://www.ualberta.ca/~fyeh/popgene_download.html). Association analysis between the mutations of the *MTHFR* genotypes and milk yield, as well as the fat and protein contents, was performed using repeated measures in the general linear model procedure of SPSS version 16.0 statistical software (http://en.softonic.com/s/spss-16-software). The mixed linear model was described by: *Y*_ikm_ = μ + *G*_i_ + *N*_k_ *+ E*_ikm_, where *Y*_ikm_ is the trait measured in each of the ikm^th^ female goat, μ is the overall population mean, *G*_i_ is the fixed effect associated with the i^th^ genotype, *N*_k_ is the fixed effect associated with the k^th^ female goat’s litter size and *E*_ikm_ is the random error. Multiple comparisons of the mean milk yield, as well as mean fat and protein contents, for different genotypes were performed via Fisher’s least significant difference (LSD) test by SPSS version 16.0. Fisher’s LSD test is initially similar to the Bonferroni multiple comparison test. It takes the square root of the Residual Mean Square from the analysis of variance and considers that to be the pooled standard deviation. Taking into account the sample sizes of the two groups being compared, it computes a standard error of the difference between those two means. Then it computes a *t* ratio by dividing the difference between means by the standard error of that difference. The effects associated with the farm, birth year and season of birth were not included in the linear model because preliminary statistical analyses indicated that these effects did not significantly influence the variability of traits in the analysed populations.

### Bioinformatics analysis of *MTHFR* 3'UTR

Bioinformatics analysis was used to predict the effects of mutations in the 3'UTR of *MTHFR* gene on the miRNA binding sites. The analysis was performed with five bioinformatics tools, namely, TargetScan(http://www.targetscan.org/), MicroInspector(http://bioinfo1.uni-plovdiv.bg/cgi-bin/microinspector/), miRWalk(http://www.umm.uni-heidelberg.de/apps/zmf/mirwalk/index.html), RNAhybrid(http://bibiserv.techfak.uni-bielefeld.de/rnahybrid/submission.html) and Segal Lab(http://genie.weizmann.ac.il/pubs/mir07/mir07_prediction.html). The combination of these approaches was considered to reduce the possibility of false positives.

### RNA isolation and real-time quantitative PCR (RT-qPCR)

To detect the correlation between expression levels of the *MTHFR* mRNA and SNPs (g.2244A>G and g.2264A>G) in vivo, mammary alveolar was stripped from 31 mammary gland tissues with different haplotypes (G-G, *n* = 12; G-A, *n* = 5; A-G, *n* = 4;A-A, *n* = 10) for RNA extraction. Total RNA was extracted from these samples using TRIzol Reagent (Invitrogen, Carlsbad, USA). The extracted RNA was homogenised for RT-qPCR. The quantity and integrity of each RNA sample were assessed with an Agilent 2100 Bioanalyser (Agilent Technologies, USA). A total of 31 RNA samples, which included the homozygous haplotypes G-G, G-A, A-G and A-A, were subjected to reverse transcription using a cDNA High Capacity Kit (Invitrogen, Carlsbad, USA). RT-qPCR analysis with the SYBR Green PCR Master Mix (Takara, Dalian, China) was performed on a CFX Connect Real-time PCR Detection System (Bio-Rad, CA, USA). The relative expression levels of objective mRNAs were calculated using the ∆∆Ct method [17]. The primers of the *MTHFR*, *UXT*, *RPS9* and *RPS15* genes are described in Table B in S1 File. The fold changes were normalised by the expression levels of *UXT*, *RPS9* and *RPS15*, and each assay was performed in triplicate [18, 19].

### Cell culture

Goat mammary epithelial cells (GMECs) were obtained from mammary gland biopsy of four 2-year-old (50 days post-parturition) Guanzhong dairy goats. Four genotype cells (GG-GG, AA-GG, GG-AA and AA-AA) were directly isolated from Guanzhong dairy goats with these genotypes. After mammary tissues were surgically removed from the dairy goats, they were placed in a sterile ice-cold D-Hank’ssolution supplemented with 300 U/mL penicillin and were immediately transported to the laboratory. The mammary tissues were trimmed of visible fat and connective tissues and washed with D-Hank’s solution several times until the solution became pellucid and devoid of milk. Tissue sections were then minced into about 1 mm^3^cubes and rinsed again with D-Hank’s solution and kept up for 10 min at room temperature. The smaller pieces of tissues were put onto the cell dishes coated with collagen and were reversely incubated at 37°C with saturated humidity and 5% CO2. After 30 min,2 mL culture medium was added to the culture dish. The culture medium consisted of DMEM-F12 supplemented with 10% FBS, insulin (5 μg/ml), hydrocortisone (0.25 μmol/L), penicillin (100 U/mL), streptomycin (100μg/mL) and epidermal growth factor 1 (10 ng/ml). The medium was replaced with fresh medium every 48 h until the cells were migrated out of the tissue and visibly spread across the bottom of the dish. Being cultured for about five passages, GMECs were obtained according to Lin et al. (2013) [20].

### Luciferase reporter assays

To construct the luciferase reporter plasmids containing the *MTHFR* 3'UTR, fragments of the *MTHFR* 3' UTR with different haplotypes (A-G, G-A, A-A and G-G) were amplified by PCR. The PCR products of the different haplotypes (A-G, G-A, A-A and G-G) were isolated and separated by agarose gel electrophoresis. These products were then linked to a pMD19-T vector with a TA Cloning Kit (Invitrogen, CA, USA). The recombinant pMD19-T vectors with different haplotypes were digested by the *Xho*I and *Not*I endonuclease enzymes. Finally, the digested products with different haplotypes were inserted between the *Renilla* and firefly luciferase genes in a psiCHECK-2 vector (Promega, WI, USA).The plasmids containing the A-G, G-A, A-A and G-G haplotypes were obtained and then confirmed by sequencing. For the luciferase reporter assay, GMECs were placed in 12-well plates (5 × 10^5^ cells per well) and then cotransfected with psiCHECK-2 vectors containing the 3'UTR-haplotypes A-G, G-A, A-A and G-G. The mimics of miRNAs (hsa-miR-1266, hsa-miR-1289, hsa-miR-505 and hsa-miR-616) and their negative controls (GenePharma, Shanghai, China) were cotransfected with the reporter plasmids to a final concentration of 20 nmol/μL. At 36 h after transfection in GMECs, *Renilla* luciferase activity in lysates was measured with the Dual-Luciferase Reporter Assay System (Promega, WI, USA).The results were normalised against firefly luciferase activity. Assays were performed according to the manufacturer’s suggestions. Each experiment was independently performed three times, and each sample was evaluated in triplicate. The Mann–Whitney test was used to compare data, and a two-sided *P* value of 0.05 was considered significant.

## Results

### SNP identification and genotyping

**Fig 1:**
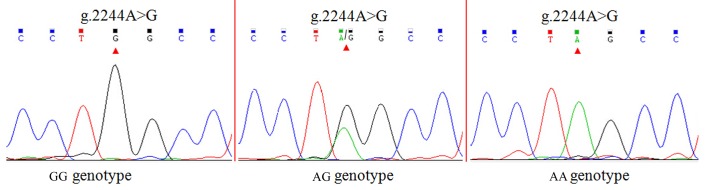
Sequencing chromatograms of different genotypes at g.2244A>G locus.

**Fig 2:**
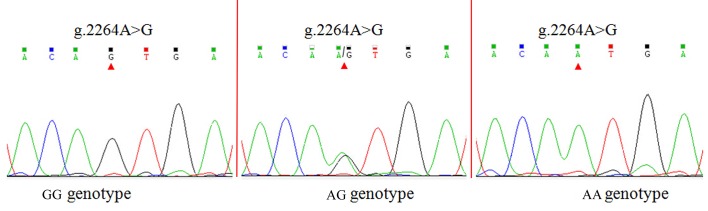
Sequencing chromatograms of different genotypes at g.2264A>G locus.

Two SNPs (g.2244A>G and g.2264A>G) were identified in the caprine *MTHFR* 3'UTRby DNA sequencing (Figs 1 and 2). The mutant sequence of *MTHFR* 3'UTR was submitted to NCBI (GenBank Accession No. KM243021). Four alleles of the g.2244A>G and g.2264A>G SNPs introduced several different miRNA sites (S1 Fig). The PIC was 0.37 at the g.2244A>G and g.2264A>G loci, respectively (Table C in S1 File). The genotypic distribution and allele frequencies of both SNPs are shown in Table C in S1 File. The g.2244A>G and g.2264A>G loci were in Hardy–Weinberg disequilibrium (*P* < 0.05) (Table C in S1 File). To reveal the linkage relationships between both SNPs, the linkage disequilibrium was estimated in the GD goats (Table C in S1 File). When *r* ^*2*^ > 0.33, the linkage disequilibrium was considered strong [21]. Thus, the g.2244A>G and g.2264A>G loci were closely linked in the GD goats.

### Association analysis of SNPs with milk production traits

Individual GD goats with AA and AG genotypes had greater milk yield than those with the GG genotype at the g.2244A>G locus (*P* < 0.05; Table D in S1 File). In addition, individuals with the AA genotype at the g.2244A>G locus had higher milk protein levels than those with the AG and GG genotypes (*P* < 0.05). The combined effects of the two SNPs (g.2244A>G and g.2264A>G) showed that individuals with the AA-GG (homozygous haplotype A-G) genotype had higher milk yield than individuals with the AA-AA (homozygous haplotype A-A), GG-AA (homozygous haplotype G-A), GG-GG (homozygous haplotype G-G) and AG-AG genotypes (*P* < 0.05; Table 1). In addition, individuals with the AA-GG (homozygous haplotype A-G) genotype had higher milk protein content than individuals with the AG-AG and GG-GG (homozygous haplotype G-G) genotypes (*P* < 0.05).

**Table 1:** Combined effect of two loci on milk production traits (means ± standard errors) in goats.

Combined genotype	Number	Haplotype	Milk yield (kg)	Milk fat (%)	Milk protein (%)
AA-AA	65	Homozygous A-A	655.96±6.21^ab^	3.38±0.07	3.07±0.02^ab^
AG-AA	6		690.45±20.45^bc^	3.70±0.13	3.07±0.04^ab^
GG-AA	7	Homozygous G-A	654.09±18.93^ab^	3.50±0.19	3.06±0.05^ab^
AG-AG	107		659.52±4.84^b^	3.45±0.04	3.02±0.02^a^
GG-AG	5		674.68±22.40^abc^	3.39±0.22	2.97±0.06^ab^
AA-GG	22	Homozygous A-G	705.52±10.68^c^	3.49±0.06	3.11±0.02^b^
GG-GG	113	Homozygous G-G	645.56±4.71^a^	3.50±0.03	3.02±0.01^a^

Note: Values with different superscripts (a, b, c, ab, abc) within the same column differ significantly at *P*< 0.05.

### Effects of SNPs on miRNA binding ability

**Fig 3:**
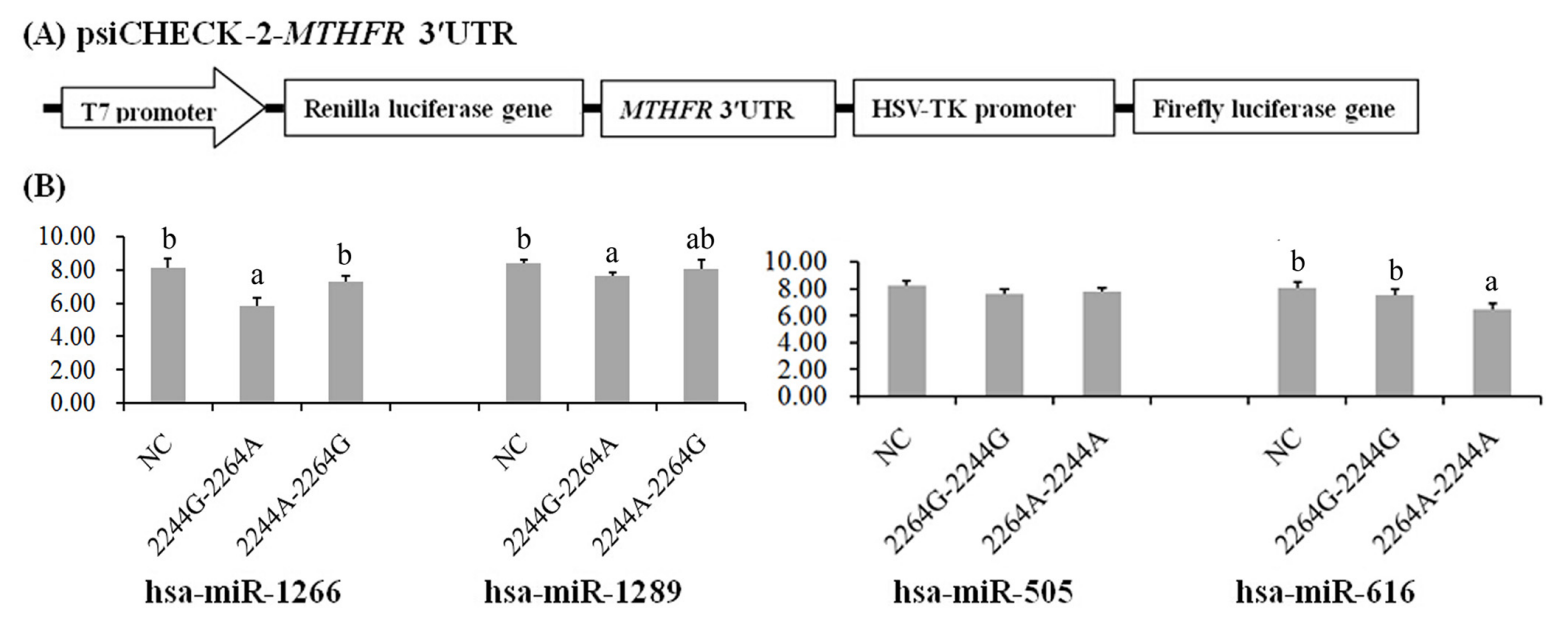
Characterization and functional analysis of *MTHFR* 3′UTR. (A) Double fluorescein enzyme expression vector linked with *MTHFR* 3′UTR. (B) GMECs cells seeded on 12-well plates were transiently cotransfected with psiCHECK-2-*MTHFR* 3′UTR and miRNA mimics (hsa-miR-1266, hsa-miR-1289, hsa-miR-505 and hsa-miR-616) or stable negative control (NC). Luciferase activity was measured after 36 h of transfection. Renilla luciferase was normalized with firefly luciferase activity, and the mean activities ± standard deviation from 3 independent experiments are shown. *P<0.05.

The miRNA target site predictions showed that hsa-miR-1266, hsa-miR-1289, hsa-miR-505 and hsa-miR-616 could bind to the 3'UTR region of the *MTHFR* gene (S2 Fig). To understand the functional significance of the g.2244A>G and g.2264A>G substitutions, we used a luciferase reporter assay (Fig 3A). Compared with the haplotype 2244A-2264G and negative control (NC), hsa-miR-1266 suppressed luciferase expression in the presence of the haplotype 2244G-2264A (*P* < 0.05; Fig 3B). Additionally, hsa-miR-1289 significantly suppressed luciferase expression in the presence of the haplotype 2244G-2264A compared with NC (*P* < 0.05), but the difference with the haplotype 2244A-2264G was not significant (*P* > 0.05; Table E in S1 File). The presence of hsa-miR-616 efficiently reduced luciferase expression in the 2264A-2244A-containing plasmid compared with that in the 2264G-2244G-containing plasmid (*P* < 0.05). However, hsa-miR-505 did not significantly affect luciferase expression (*P* > 0.05; Table F in S1 File). These results suggested that the haplotypes 2244G-2264A and 2264A-2244A had lower MTHFR protein expression levels than the haplotype 2244A-2264G and 2264G-2244G.

**Fig 4:**
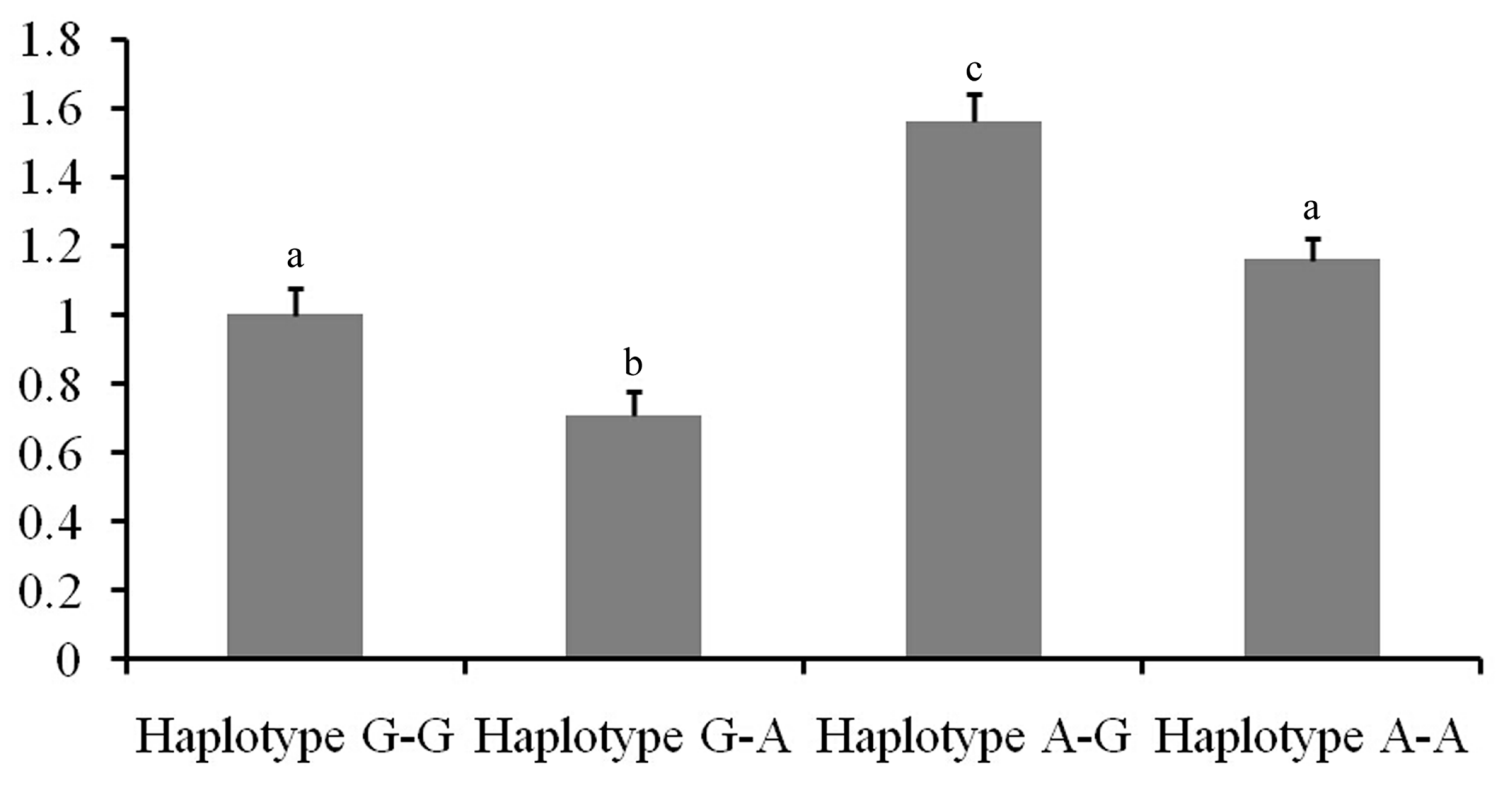
Comparison of mRNA expression levels of caprine *MTHFR* in mammary glands among four haplotypes. The number of animals of homozygous haplotypes G-G, G-A, A-G and A-A were 12, 5, 4 and 10, respectively. The fold change was normalized against *UXT*, *RPS9and RPS15*genes. The different small letters represent significant difference at 5% level.

In this study, we performed RT-qPCR assays to explore whether hsa-miR-1266 and hsa-miR-616 affect MTHFR expression by degrading mRNA or suppressing mRNA post-translational translation (Fig 4). A total of 31 mammary gland samples with different haplotypes for the *MTHFR*g.2244A>G and g.2264A>G polymorphisms were used to assess the expression of *MTHFR* mRNA. Compared with individuals with homozygous haplotype G-G, A-G and A-A, the individuals with homozygous haplotype G-A had the lowest mRNA expression. By contrast, individuals with homozygous haplotype A-G had the highest mRNA expression among all the samples (Table G in S1 File). Therefore, hsa-miR-1266 and hsa-miR-616 may affect *MTHFR* mRNA expression by degrading mRNA.

## Discussion

PIC is related to the use efficiency and selection potential of genetic markers; the greater polymorphism information content values and heterozygous ratio are, the greater the potentials of genetic markers are, and their effects are better for animal genetic breeding [16]. PIC values are classified as low polymorphism when PIC < 0.25, moderate polymorphism when 0.25 < PIC < 0.50 and high polymorphism when PIC > 0.50). The GD goats have moderate genetic diversity at the g.2244A>G and g.2264A>G loci, so the both loci have a better potential as genetic markers.

The 3'UTR polymorphisms may be within or at the vicinity of the miRNA binding site; these polymorphisms may interfere with miRNA function and lead to differential gene expression, thereby affecting the animal phenotype [22]. Previous reports showed that such polymorphisms influence *MTHFR* gene expression [23, 24]. The human *MTHFR* rs4846049 (G>T) is a potential candidate SNP that modulates miRNAs; the *MTHFR* mRNA complex has the greatest change in the binding free energy for hsa-miR-149; based on luciferase analysis, hsa-miR-149 inhibited the activity of the reporter vector carrying the T allele, but not the G allele [25]. The effects of SNPs in the 3' UTR of *MTHFR* on animal production traits have remained largely unknown. In the present study, the results of our integrative analysis showed that the g.2244A>G and g.2264A>G SNPs may change the binding activity of miRNAs (hsa-miR-1266 and hsa-miR-616) with the *MTHFR* 3'UTR. We used a bioinformatics analysis method similar to that of Sun et al. [26], who reported that the transcribed target SNPs associated with schizophrenia alter the binding activity of miRNAs with the 3'UTR of mRNA. Therefore, we speculated that the SNPs g.2244A>G and g.2264A>G accounted for differences in the milk production traits of GD goats because hsa-miR-1266 and hsa-miR-616 could change the expression levels of the *MTHFR* gene.

The results of the association analysis showed that individuals with the homozygous haplotype A-G had higher milk yield and protein content than those with other haplotypes. Functional assays indicated that the *MTHFR* 2244 A → G substitution could increase the binding activity of hsa-miR-1266 with the *MTHFR* 3'UTR, whereas the *MTHFR* 2264 A → G substitution could decrease the binding activity of hsa-miR-616 with the *MTHFR*3'UTR. These data were consistent with results of *in silico* analysis, thereby suggesting the functional interaction between hsa-miR-1266 or hsa-miR-616 and the *MTHFR* mRNA 3' UTR. We further examined the *MTHFR* mRNA levels in GD goats with different haplotypes. A significant increase in the *MTHFR* mRNA levels of the homozygous haplotype A-G carriers was observed relative to those of the homozygous haplotype G-A carriers. These findings consistently suggested that the g.2244A>G and g.2264A>G SNPs altered the *MTHFR* expression level, and they were associated with milk production traits. However, this study had several limitations. Firstly, the implied regulation of *MTHFR* expression by hsa-miR-1266 and hsa-miR-616 is based on preliminary results. Further functional experiments are required to analyse the effects of this polymorphism on *MTHFR* function. Secondly, the influence of other miRNAs on mutant forms of the *MTHFR* gene must be considered.

In conclusion, our data suggested that the g.2244A>G and g.2264A>G polymorphisms were associated with milk production traits in GD goats. Future investigations should explore the underlying miRNA-mediated mechanisms that are modified by the SNPs g.2244A>G and g.2264A>G. This study evaluated these SNPs as genetic markers for goat genetics and breeding, with potential applications in breeding programs.

## Supporting Information

S1 Fig Effects of SNPs g.2244A>G (A) and g.2264A>G (B) on miRNA binding sites in the caprine *MTHFR* 3MTHFR. The green boxes represent potential miRNA target sites.
(TIF)

S2 Fig Sequence of the heptamer motif for each miRNA target site. Green represents miRNA. Red represents *MTHFR* 3THFR.
(TIF)

S1 File Analysis results of primer information, genotypic distribution, association of SNPs with milk production traits, luciferase activity, *MTHFR* mRNA expression levels and miRNA target sites. Primer information of *MTHFR* gene (**Table A**). Primer information for real-time quantitative PCR (**Table B**). Genotypic distribution and allelic frequencies of two SNP loci in the caprine *MTHFR* gene (**Table C**). Association analysis of g.2244A>G and g.2264A>G loci with milk production traits (means ± standard errors) in Guanzhong dairy goats (**Table D**). Luciferase activity (means ± standard deviation) of the psiCHECK-2-MTHFR 3′UTR vector that include haplotypes 2244G-2264A and 2244A-2264G (**Table E**). Luciferase activity (means ± standard deviation) of the psiCHECK-2-MTHFR 3′UTR vector that include haplotypes 2264G-2244G and 2264A-2244A (**Table F**). Comparison of expression levels of caprine *MTHFR* mRNA in mammary glands among four haplotypes (**Table G**).
(DOC)
