# Supplementary figures and images for: Two Mutations in the Caprine MTHFR 3'UTR Regulated by MicroRNAs Are Associated with Milk Production Traits
Source: PLoS One. 2015 Jul 17;10(7):e0133015. doi: 10.1371/journal.pone.0133015 (PMC4505847; doi:10.1371/journal.pone.0133015)

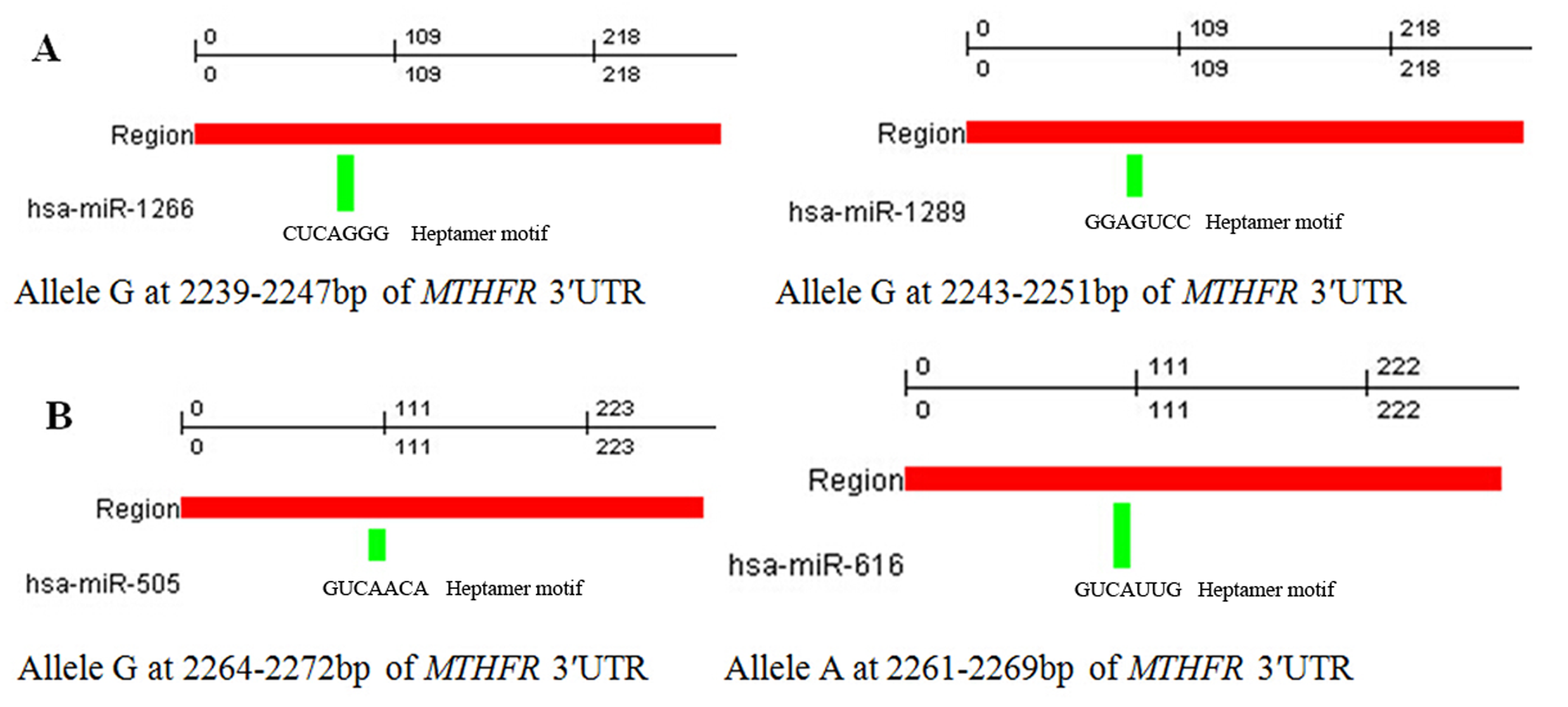

Supplement: S1 Fig — The green boxes represent potential miRNA target sites. (TIF) [file pone.0133015.s001.tif]

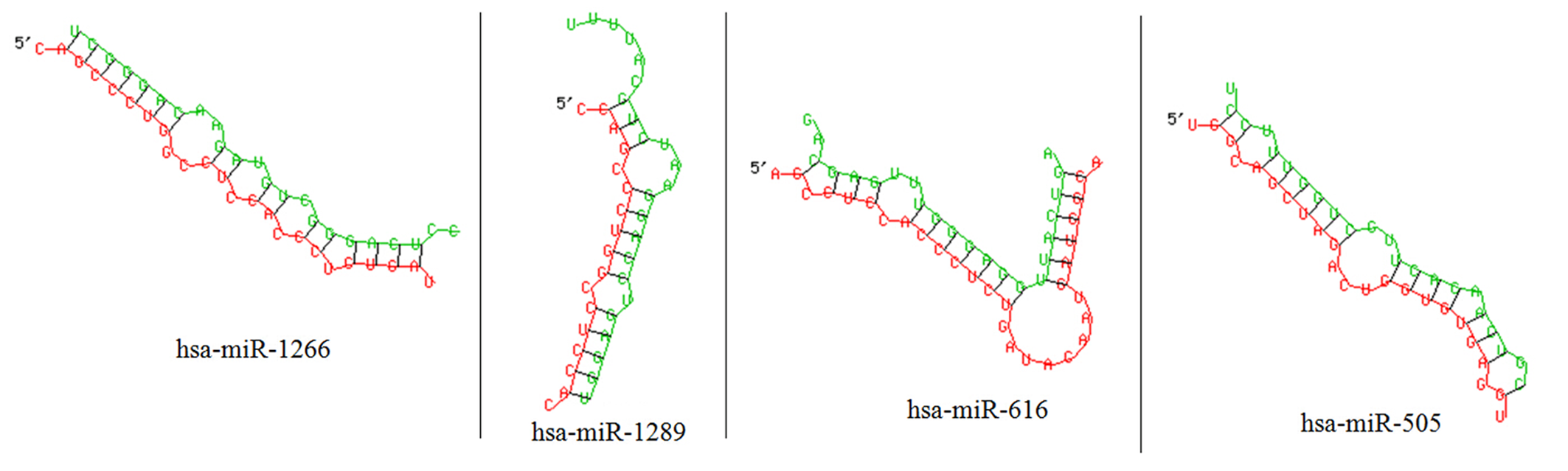

Supplement: S2 Fig — Green represents miRNA. Red represents MTHFR 3THFR. (TIF) [file pone.0133015.s002.tif]
