# Supplementary material for: Two Mutations in the Caprine MTHFR 3'UTR Regulated by MicroRNAs Are Associated with Milk Production Traits
Source: PLoS One. 2015 Jul 17;10(7):e0133015. doi: 10.1371/journal.pone.0133015 (PMC4505847; doi:10.1371/journal.pone.0133015)
Supplement: S1 File — Primer information of MTHFR gene (Table A). Primer information for real-time quantitative PCR (Table B). Genotypic distribution and allelic frequencies of two SNP loci in the caprine MTHFR gene (Table C). Association analysis of g.2244A>G and g.2264A>G loci with milk production traits (means ± standard errors) in Guanzhong dairy goats (Table D). Luciferase activity (means ± standard deviation) of the psiCHECK-2-MTHFR 3′UTR vector that include haplotypes 2244G-2264A and 2244A-2264G (Table E). Luciferase activity (means ± standard deviation) of the psiCHECK-2-MTHFR 3′UTR vector that include haplotypes 2264G-2244G and 2264A-2244A (Table F). Comparison of expression levels of caprine MTHFR mRNA in mammary glands among four haplotypes (Table G). (DOC) [file pone.0133015.s003.doc]

**Supporting Information**

**Table A. Primer information of** ***MTHFR* gene for detecting SNP and cloning 3′UTR.**

| Primer | Sequence (5'→3') | Objective | GenBank accession No. XM_005690674 | Product  size (bp) | Ta (^o^C) |
| --- | --- | --- | --- | --- | --- |
| MF1 | GCCTTCCTCATCTCCATC | Screening polymorphism | 2185-2202 | 326 | 50 |
| MR1 | CACTTTGCCTGTTCTCCT |  | 2493-2510 |  |  |
| MF2 | CTCGAGGCCTTCCTCATCTCCATC | Cloning 3′UTR | 2185-2202 | 340 | 52 |
| MR2 | GCGGCCGCCACTTTGCCTGTTCTCCT |  | 2493-2510 |  |  |

Note: Ta = annealing temperature. The underlined bases in MF2/KR2 represent *Xho*I and *Not*I endonuclease enzyme loci.

**Table B. Primer information for real-time quantitative PCR.**

| GenBank accession No. | Gene | Sequence (5'→3') | Region | Product  size (bp) | Ta (^o^C) | E |
| --- | --- | --- | --- | --- | --- | --- |
| XM_005690674 | *MTHFR* | F: CCAGGGTGCTGTCAATCTCA | 382 | 80 | 60 | 1.96 |
|  |  | R: GGTGCCAGGTCACGTCTACA | 461 |  |  |  |
| XM_005700842 | *UXT* | F: GGACCATCGCGACAAGGTAT | 108 | 74 | 60 | 1.95 |
|  |  | R: CCTGGAGTCGCTCAATGACA | 181 |  |  |  |
| XM_005709411 | *RPS9* | F: AGCTGCTGACGCTGGATGA | 176 | 62 | 60 | 1.96 |
|  |  | R: CCCCAACAGGGCATTACCT | 237 |  |  |  |
| XM_005709427 | *RPS15* | F: CTGCGCGACATGATCATTCT | 295 | 59 | 60 | 1.97 |
|  |  | R: CCGTTGTAGACGCCAACCAT | 353 |  |  |  |

*Note*: The amplification efficiency (E) of primers was calculated as follow: E = 10^-1/slope^; the slope was obtained by the 7 points standard curve, with a minimum *r^2^* of 0.99.

**Table C. Genotypic distribution and allelic frequencies of two SNP loci in the caprine *MTHFR* gene.**

| Locus |  | | Number of animals |
| --- | --- | --- | --- |
| g.2244A>G | Genotype | GG | 125 |
|  |  | AG | 113 |
|  |  | AA | 87 |
|  | Allele | G | 0.56 |
|  |  | A | 0.44 |
|  | He | | 0.35 |
|  | PIC | | 0.37 |
|  | Equilibrium  χ^2^ test | | *P*<0.01 |
| g.2264A>G | Genotype | GG | 135 |
|  |  | AG | 112 |
|  |  | AA | 78 |
|  | Allele | G | 0.59 |
|  |  | A | 0.41 |
|  | He |  | 0.34 |
|  | PIC |  | 0.37 |
|  | Equilibrium  *χ*^2^ test |  | *P*<0.01 |
| LD of g.2244A>G and g.2264A>G |  |  | *r^2^*=0.60 |

Note: LD = linkage disequilibrium

**Table D. Association analysis of g.2244A>G and g.2264A>G loci with milk production traits (means ± standard errors) in Guanzhong dairy goats.**

| Locus | Genotype | Milk yield (kg) | Milk fat (%) | Milk protein (%) |
| --- | --- | --- | --- | --- |
| g.2244A>G | AA(87) | 668.49±5.50^b^ | 3.41±0.05 | 3.08±0.02^b^ |
|  | AG(113) | 661.16±4.83^b^ | 3.46±0.04 | 3.03±0.02^a^ |
|  | GG(125) | 647.21±4.59^a^ | 3.50±0.03 | 3.02±0.01^a^ |
| g.2264A>G | AA(78) | 658.45±5.89 | 3.42±0.06 | 3.06±0.02 |
|  | AG(112) | 660.20±4.92 | 3.45±0.02 | 3.02±0.02 |
|  | GG(135) | 655.33±4.48 | 3.50±0.03 | 3.04±0.01 |

Note: Values with different superscripts within the same column differ significantly at *P* < 0.05.

**Table E. Luciferase activity (means ± standard deviation) of the psiCHECK™-2-MTHFR 3′UTR vector that include haplotypes 2244G-2264A and 2244A-2264G.**

| miRNA | Group | Luciferase  activity | *P*-values | | |
| --- | --- | --- | --- | --- | --- |
|  |  |  | Negative control | 2244G-2264A | 2244A-2264G |
| hsa-miR-1266 | Negative control | 8.12±0.51^b^ | - |  |  |
|  | 2244G-2264A | 5.86±0.48^a^ | 0.001 | - |  |
|  | 2244A-2264G | 7.26±0.38^b^ | 0.060 | 0.010 | - |
| hsa-miR-1289 | Negative control | 8.39±0.21^b^ | - |  |  |
|  | 2244G-2264A | 7.61±0.24^a^ | 0.039 | - |  |
|  | 2244A-2264G | 8.05±0.54^ab^ | 0.288 | 0.195 | - |

Note: Values with different superscripts within the same column differ significantly.

.

**Table F. Luciferase activity (means ± standard deviation) of the psiCHECK™-2-MTHFR 3′UTR vector that include haplotypes 2264G-2244G and 2264A-2244A.**

| miRNA | Group | Luciferase  activity | *P*-values | | |
| --- | --- | --- | --- | --- | --- |
|  |  |  | Negative control | 2264G-2244G | 2264A-2244A |
| hsa-miR-505 | Negative control | 8.26±0.43 | - |  |  |
|  | 2264G-2244G | 7.59±0.33 | 0.064 | - |  |
|  | 2264A-2244A | 7.75±0.32 | 0.138 | 0.601 | - |
| hsa-miR-616 | Negative control | 8.01±0.45^b^ | - |  |  |
|  | 2264G-2244G | 7.49±0.42^a^ | 0.169 | - |  |
|  | 2264A-2244A | 6.48±0.36^a^ | 0.004 | 0.024 | - |

Note: Values with different superscripts within the same column differ significantly.

.

**Table G. Comparison of expression levels of caprine *MTHFR* mRNA in mammary glands among four haplotypes.**

| Haplotype | Relative expression  level | *P*-values | | | |
| --- | --- | --- | --- | --- | --- |
|  |  | Haplotype G-G | Haplotype G-A | Haplotype A-G | Haplotype A-A |
| Haplotype G-G | 1.00±0.07^a^ | - |  |  |  |
| Haplotype G-A | 0.73±0.10^b^ | 0.001 | - |  |  |
| Haplotype A-G | 1.60±0.12^c^ | 0.000 | 0.000 | - |  |
| Haplotype A-A | 1.14±0.15^a^ | 0.083 | 0.000 | 0.001 | - |

Note: Values with different superscripts within the same column differ significantly.
